# The GAAIN Entity Mapper: An Active-Learning System for Medical Data Mapping

**DOI:** 10.3389/fninf.2015.00030

**Published:** 2016-01-13

**Authors:** Naveen Ashish, Peehoo Dewan, Arthur W. Toga

**Affiliations:** Laboratory of Neuro Imaging, Keck School of Medicine, Stevens Neuroimaging and Informatics Institute, University of Southern CaliforniaLos Angeles, CA, USA

**Keywords:** data mapping, machine learning, active Learning, data harmonization, common data model

## Abstract

This work is focused on mapping biomedical datasets to a common representation, as an integral part of data harmonization for integrated biomedical data access and sharing. We present GEM, an intelligent software assistant for automated data mapping across different datasets or from a dataset to a common data model. The GEM system automates data mapping by providing precise suggestions for data element mappings. It leverages the detailed metadata about elements in associated dataset documentation such as data dictionaries that are typically available with biomedical datasets. It employs unsupervised text mining techniques to determine similarity between data elements and also employs machine-learning classifiers to identify element matches. It further provides an active-learning capability where the process of training the GEM system is optimized. Our experimental evaluations show that the GEM system provides highly accurate data mappings (over 90% accuracy) for real datasets of thousands of data elements each, in the Alzheimer's disease research domain. Further, the effort in training the system for new datasets is also optimized. We are currently employing the GEM system to map Alzheimer's disease datasets from around the globe into a common representation, as part of a global Alzheimer's disease integrated data sharing and analysis network called GAAIN^1^. GEM achieves significantly higher data mapping accuracy for biomedical datasets compared to other state-of-the-art tools for database schema matching that have similar functionality. With the use of active-learning capabilities, the user effort in training the system is minimal.

## Background and significance

This paper describes a software solution for biomedical data *harmonization*. Our work is in the context of the “GAAIN” project in the domain of Alzheimer's disease data. However, this solution is applicable to any biomedical or clinical data harmonization in general. GAAIN—the Global Alzheimer's Association Interactive Network is a data sharing federated network of Alzheimer's disease datasets from around the globe. The aim of GAAIN is to create a network of Alzheimer's disease data, researchers, analytical tools and computational resources to better our understanding of this disease. A key capability of this network is also to provide investigators with access to harmonized data across multiple, independently created Alzheimer's datasets.

**Figure 1:**
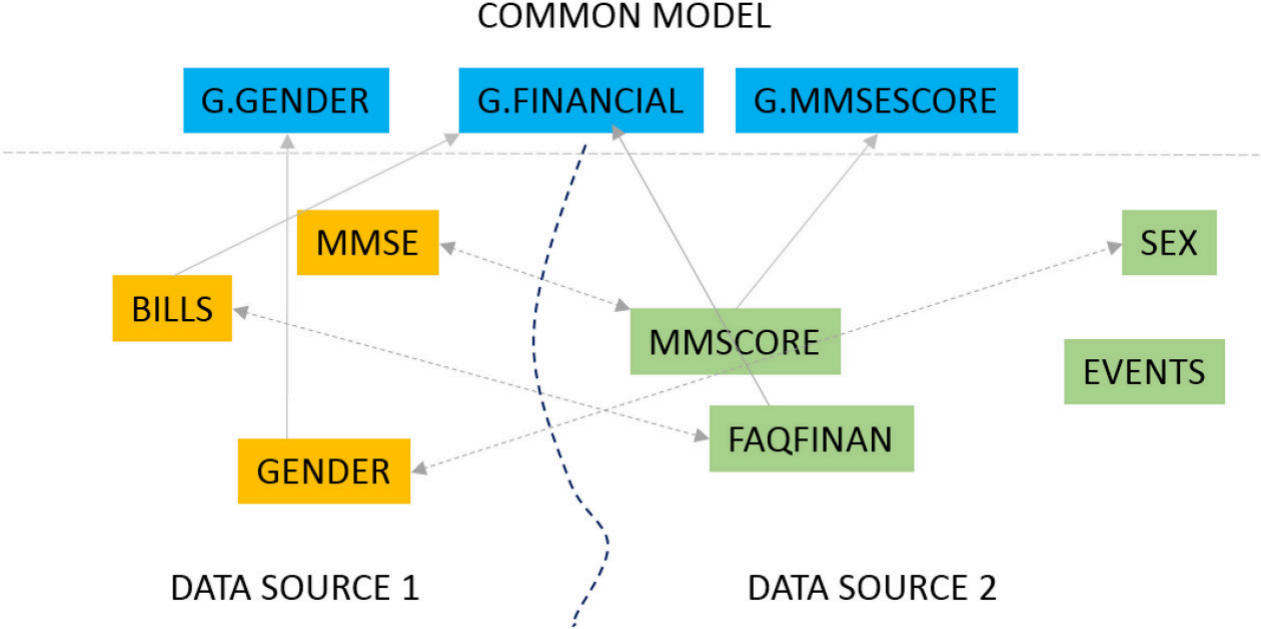
**Data element mapping**.

Our primary interest is in biomedical data sharing and specifically harmonized data sharing. Harmonized data from multiple data providers has been curated to a unified representation after reconciling the different formats, representation, and terminology from which it was derived (Doan et al., 2012, Principles of data integration; Ohmann and Kuchinke, 2009). The process of data harmonization can be resource intensive and time consuming the present work describes a software solution to significantly automate that process. Data harmonization is fundamentally about data alignment - which is to establish correspondence between related or identical data elements across different datasets. Consider the very simple example of a data element capturing the gender of a subject that is defined as “SEX” in one dataset, “GENDER” in another and “M/F” in yet another. When harmonizing data, a unified element is needed to capture this gender concept and to link (align) the individual elements in different datasets with this unified element. This unified element is the “G.GENDER” element as illustrated in Figure 1.

The data mapping problem can be solved in two ways as illustrated in Figure 1. We could map elements across two datasets, for instance match the element “GENDER” from one data source (DATA SOURCE 1 in Figure 1) to the element “SEX” in a second source (DATA SOURCE 2). We could also map elements from one dataset to elements from a *common data model*. A common data model is a uniform representation which all data sources or providers in a data sharing network agree to adopt. The fundamental mapping task is the same in both. Also, the task of data alignment is inevitable regardless of the data sharing model one employs. In a *centralized data sharing* model (NDAR, 2015), where we create a single unified store of data from multiple data sources, the data from any data source must be mapped and transformed to the unified representation of the central repository. In *federated* or *mediated* approaches to data sharing (Doan et al., 2012, Principles of data integration) individual data sources (such as databases) must be mapped to a “global” unified model through mapping rules. The common data model approach, which is also the GAAIN approach, also requires us to map and transform every dataset to the (GAAIN) common data model. This kind of data alignment or mapping can be labor intensive in biomedical and clinical data integration case studies (Ashish et al., 2010). A single dataset typically has thousands of distinct data elements of which a large subset must be accurately mapped. And it is widely acknowledged that data sharing and integration processes need to be simplified and made less resource intensive for data sharing, for them to become a viable solution for the medical and clinical data sharing domain as well as the more general enterprise information integration domain (Halevy et al., 2005). The GEM system is designed to achieve this by providing automated assistance to developers for such data alignment or mapping.

The GEM data mapping approach is centered on exploiting the information in the data documentation, typically in the form of *data dictionaries* associated with the data. The importance of data dictionary documentation, and for Alzheimer's data in particular, has been articulated in Morris et al. (2006). These data dictionaries contain detailed descriptive information and metadata about each data element in the dataset. Our solution is based on extracting this rich metadata in data dictionaries, developing element similarity measures based on text mining of the element descriptions, and employing machine-learning classifiers to meaningfully combine multiple indicative factors for an element match.

## Materials and methods

Here we report the second version of the GEM system (Ashish et al., 2015). The first version (GEM-1.0) (Ashish et al., 2015), deployed in December 2014 is a knowledge driven system. Element matches are determined in a heuristic manner based on element similarity derived off the element metadata in data dictionaries. In this second version (GEM-2.0), completed in April 2015, we added machine-learning based classification to the system. We have further added capabilities of active-learning (Cohn et al., 1996) where the user effort in training the machine-learning classifiers in the system is minimized.

While GEM-2.0 data mapping is powered by machine-learning classification, it employs the element metadata extraction developed in GEM-1.0 for synthesizing features required for classification. In this section we will describe the extraction of metadata from data dictionaries and element similarity metrics as developed in GEM-1.0. We then describe the machine-learning classification capabilities developed in GEM-2.0. The last sub-section describes the active-learning capability for training the system for new datasets, in an efficient manner. Before describing the system however, we clarify some terminology and definitions.

- A dataset is a *source* of data. For example a dataset provided by “ADNI”^2^ is a source.
- A *data dictionary* is the document associated with a dataset, which defines the terms used in the dataset.
- A *data element* is an individual “atomic” unit of information in a dataset, for instance a field or a column in a table in a database or in a spreadsheet.
- The documentation for each data element in a data dictionary is called *element metadata* or *element information*.
- A *mapping* or element mapping is a one-to-one relationship across two data elements, coming from different sources.
- Mappings are created across two distinct sources. The element that we seek to match is called the *query element*. The source we must find matches *from* is called the *target source* and the source of the query element is called the *query source*.
  ° Note: A common data model may also be treated as a target source.
- For any element, the GEM system provides a set of suggestions (typically 5). We refer to this set as the *window* of suggestions.

### Metadata extraction for element similarity

Medical datasets, including datasets in domains such as Alzheimer's disease, are typically documented in data dictionaries. The data dictionary provides information about each element, including descriptions of the data element and other details such as its data type, range or set of possible values etc.

**Figure 2:**
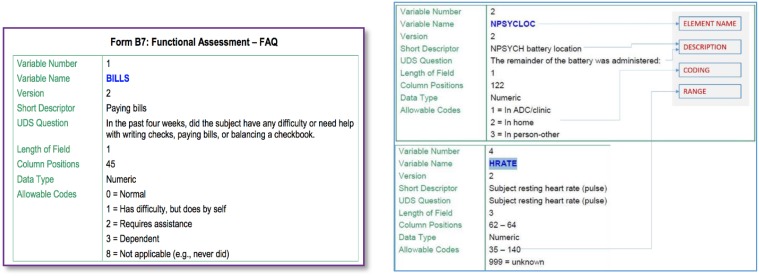
**Element information in data dictionary**.

Figure 2 illustrates some snippets from a data dictionary for a particular Alzheimer's disease dataset. The element described (on the left) is called “BILLS” and includes a short as well as a more complete description of this data element. We are also provided information about its type (numeric code in this case) and also the possible values it can take, i.e., one of {0,1,2,3,8}. Such element information is the basis for identifying GEM element mappings.

As a data mapping system GEM operates as follows:

- Elements are matched based on a metric of *element similarity* that is assessed by using the above illustrated metadata for each element.
- Such metadata is first extracted from the data dictionaries (of the datasets to be matched) and stored in a *metadata database*.
- Elements are matched from a *source* dataset to a *target* dataset. For instance we may be interested in finding mappings between two datasets such as ADNI and say “NACC”^3^ of which one would be the source and the other the target.
  ° The target may also be a common data model. The elements of the common model are treated as (data) elements of a target data model.
- In determining the possible matches for a given data element, some of the metadata constraints are used for “blocking,” i.e., elimination from consideration of improbable matches. For instance an element (such as say BILLS) that takes one of 5 distinct coded values (as illustrated above) cannot match to an element such as BLOOD PRESSURE that takes values in the range 90–140.
- Probable matches for a data element are determined using element similarity. The element similarity is a score that captures how well (or not) the corresponding text descriptions of two data elements match. This text description similarity is computed using two approaches.
  ° One is to use a *TF-IDF* cosine distance on a bag-of-words representation of the text descriptions. TF-IDF, short for term frequency–inverse document frequency, is a numerical statistic that is intended to reflect how important a word is to a document in a collection or corpus (Robertson, 2004).
  ° Another approach requires building a topic model (Blei, 2012) over the element text descriptions and using the topic distribution to derive the similarity between two element text descriptions.

In machine learning and natural language processing, a topic model is a type of statistical model for discovering the abstract topics that occur in a collection of documents (Blei, 2012). Such topics are not known in advance and are discovered dynamically during topic modeling. Topics are defined by a set of words from the document collection. After topic modeling a document is not assigned to a single topic (necessarily) but rather a document's topic is essentially a distribution over multiple topics. In the second approach for determining text similarity we compare how similar the topic distributions of two documents (element descriptions in this case) are.

**Figure 3:**
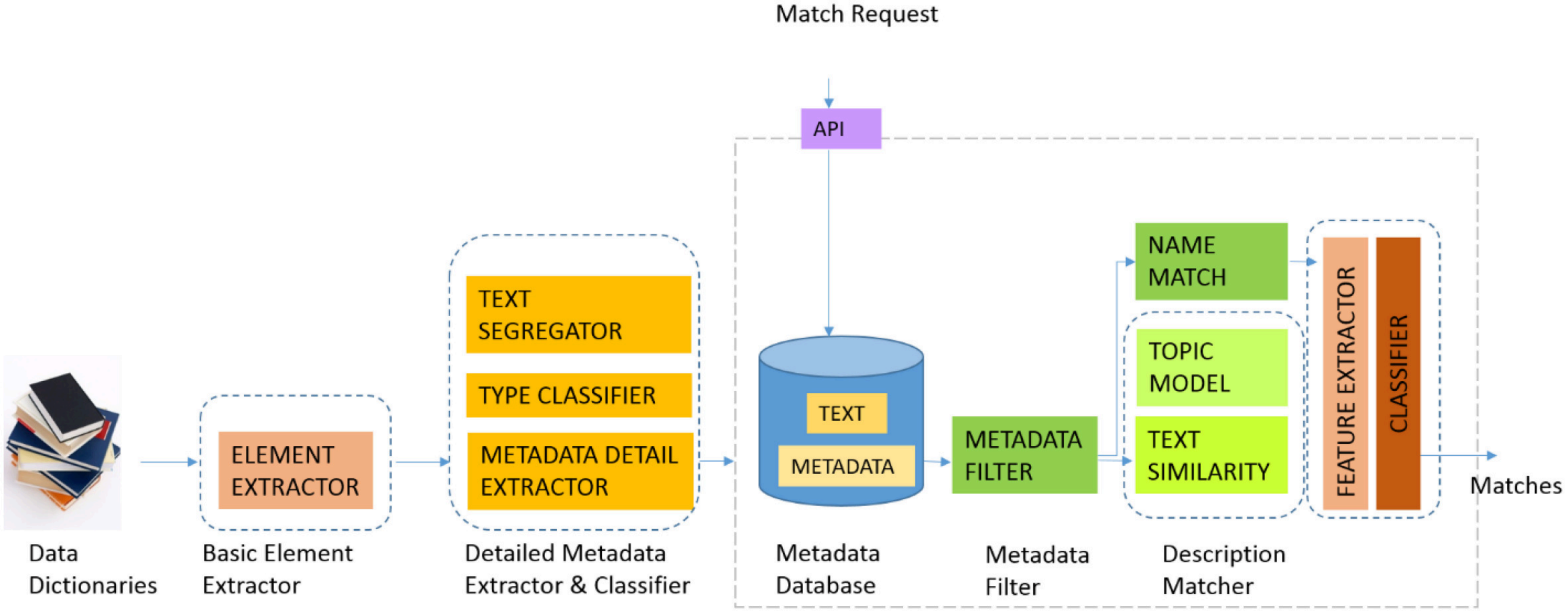
**GEM architecture**.

Figure 3 illustrates the current GEM-2.0 architecture and workflow, GEM-1.0 had the same components minus the machine-learning classification modules, namely the boxes labeled “FEATURE EXTRACTOR” and “CLASSIFIER” to the right in Figure 3. In GEM-2.0 we also added a “NAME MATCH” module that determines the similarity of two element names—this module is capable of matching complex element names common in biomedical datasets and achieves this by segmenting complex element names into “components” and matching element names in a component wise fashion. In GEM-1.0 we determined element matches by (i) Blocking or filtering out improbable match candidates based on metadata constraints (the METADATA FILTER in Figure 1) and (ii) Ranking probable matches in order of the element similarity that was based on the element text description similarity. GEM-1.0 is described in more detail in Ashish et al. (2015).

### Machine learning classification

Our motivation for the incorporation of machine-learning based classification for data mapping in GEM is two-fold. First, we have multiple indicators that can help in identifying element matches—for example text similarity measures, other metadata constraints such as the element data type or set or range of possible values, and also a measure of the similarity of element names themselves. A classification approach can learn how to optimally combine such various indicators for an element match. Next, the GEM system is intended to operate as an intelligent software assistant that suggests data mappings to human data analysts and data integration developers. As such analysts and developers “select” correct matches (from alternatives provided by the system), implicitly providing training data to the system in terms of labeled data mapping examples. The system must incorporate this training data and improve the data mapping by leveraging the new knowledge.

The classification problem in our system is to determine, given a pair of element names (from the source and target), whether they match (Y) or not (N). We now describe the features employed for this task.

#### Features for classification

The classification is driven off features that are based on the metadata extracted and the computed element similarity. Note that the features as defined for a *pair* of data elements as the classification task is to classify element pairs into whether they match or not. The features are in four broad categories:

- Features based on the element text description similarity
- Features based on the element name
- Feature based on metadata constraints
- Other features

Table 1 provides the feature set for classification where we have provided the specific features, what they essentially represent and their possible values. Many of these features are synthesized from the information extracted from the data dictionary about the elements. For instance the text description similarity scores and ranks are synthesized from the element text descriptions, this is described in more detail in Ashish et al. (2015). The name match score is provided by a name-matching classification module. Other features like the element cardinality or range are extracted directly from the data dictionary.

**Table 1:** **Set of features for classification**.

**Feature basis**	**Feature**	**Description**	**Values**
Description similarity	TFIDF^4^ based text similarity score [Tfidf score]	We calculate the similarity score of the text descriptions based on TFIDF similarity of the two data elements present in their respective data dictionaries.	A (real) value in the range 0.0–1.0
	Topic model based text similarity score [Topic score]	We build a topic model from the column descriptions of all the data elements of the two sources. We then calculate a similarity score based on the cosine similarity of the topic distributions of the two data elements.	A (real) value in the range 0.0–1.0
	TFIDF rank [Tfidf rank]	The (ordinal) rank based on the TFIDF text match score	Integer with 1 denoting the top (best) match
	Topic model rank [Topic rank]	The rank based on the topic model based text match score	Integer with 1 denoting top match
	Edit distance [Edit distance]	A word-based edit distance between associated element text descriptions	Integer
Element names	Name match applicable [Name match]	Whether a name match score is applicable (for a given element pair) or not	Binary, Y or N
	Name match score [Name score]	A name match score that is provided by an element name matching classifier module	A (real) value in the range 0.0–1.0
Metadata constraints	Cardinality [Source/Target cardinality]	The number of possible data values for an element (if discrete)	Integer ≥ 1
	Range [Source/Target min/max]	The numeric range (if applicable)	Real numbers for min and max of range
Other	Table correspondence score [Table score]	This is a score that captures how well do the tables that two elements belong to respectively, correspond to each other	A (real) value in the range 0.0–1.0

The Table Correspondence Score needs some explanation. Most datasets are originally housed in database systems, often in relational database management systems in a relational format. Data elements are thus organized and grouped into specific tables, for instance all patient information and demographic elements (patient age, gender, ethnicity etc.,) would likely be represented as columns in a demographics table. The table association can be a useful indicator for data mapping. For instance if we know that certain elements such as AGE, RACE, ETHNICITY all belong to a certain table “X” in the source dataset, and that their corresponding matching elements all (or mostly) come from a table “Y” in the target dataset, then it is likely that a new element say GENDER also from table X in the source has its corresponding matching element in table Y in the target. The literal table names of elements are not usable features, as the system would, at best, only learn about the specific table correspondences in the training data. This would not scale to unseen table names in the data that must be actually mapped. We have thus synthesized a feature called the *table correspondence score* which is a measure of how well two tables (one from the source and the other from the target) “correspond.” Employing the metadata constraints and the element text description similarity, we can determine the “best” match for a source element, this is essentially what GEM-1.0 does. The table correspondence score (TCS) captures the proportion of such best matches across data elements from two tables and is defined as:

$$TCS(e_{S},e_{T})=\frac{M(e_{S},e_{T})}{\min(O(Tab(e_{S})),O(Tab(e_{T})))}$$

where,

$$\begin{array}{c} Tab(e)\text{ is the table to which element }e\text{ belongs}\\ M(e_{S},e_{T})=\{\begin{array}{ll} 1 & \text{if best match of }e_{S}\text{ is in }Tab(e_{T})\\ 0 & \text{otherwise} \end{array}\\ O(Tab(e))=\text{size of the table of element }e\text{ (number of columns in}\\ \text{table)} \end{array}$$

The FEATURE EXTRACTOR module (Figure 3) compiles these above features from the metadata database. The CLASSIFIER module is a suite of machine-learning classifiers that can be trained on annotated training data for a pair of datasets to be mapped. We have experimented with multiple kinds of classifiers including functional classifiers such as Support Vector Machines (SVM) and Logistic Regression, tree based classifiers such as Random Forest and Simple CART, hybrid classifiers such as Sequential Minimal Optimization (SMO) and also neural network classifiers such as the Multi-Layer Perceptron (Michalski et al., 2013). A comparative analysis of the relative performance of these classifiers is provided in the Results section.

### Active learning

The GEM system is intended for use as an intelligent software assistant that provides precise mapping suggestions but it is a human analyst that finally accepts or rejects suggested element mappings. This forms the basis for active-learning in GEM where the user is also provided assistance by the system in intelligently selecting data mapping instances for training. The GEM user interface (UI) serves a dual purpose as an intelligent assistant to help with mappings, and also as an active-learning tool that collects data for training.

Active learning (Cohn et al., 1996) itself is a special case of semi-supervised machine learning in which a learning algorithm is able to interactively query the user (or some other information source) to obtain the desired outputs at new data points. There are situations in which unlabeled data is abundant but manual labeling is expensive. In such a scenario, learning algorithms can actively query the user/teacher for labels. Since the learner chooses the examples, the number of examples to learn a concept can often be much lower than the number required in normal supervised learning.

**Figure 4:**
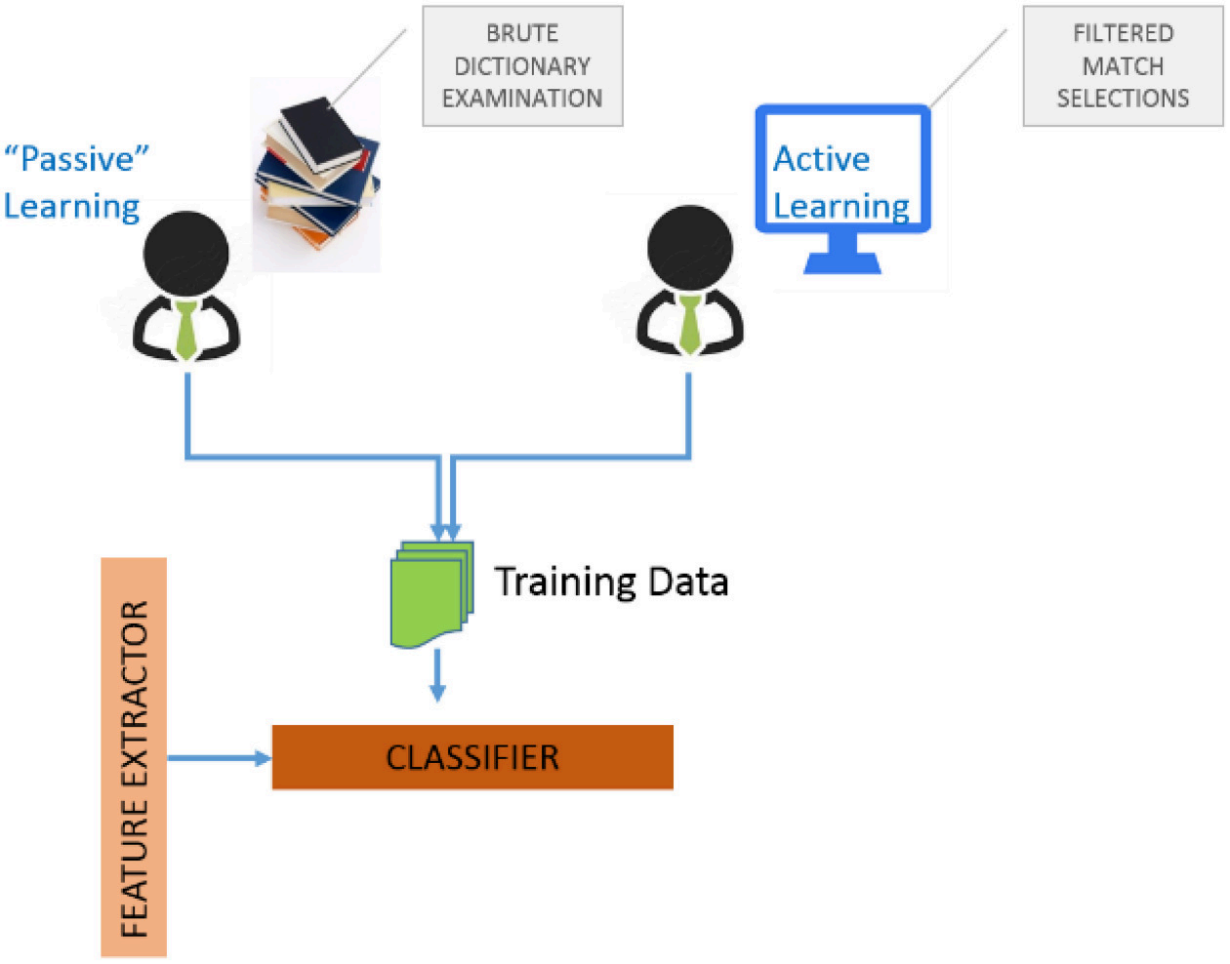
**Training data for GEM**.

Algorithms for determining which data points should be labeled can be organized into a number of different categories (Michalski et al., 2013). These include (i) uncertainty sampling where we label those points for which the current model is least certain as to what the correct output should be, (ii) query by committee where a variety of models are trained on the current labeled data, and vote on the output for unlabeled data, (iii) label those points for which the “committee” disagrees the most, (iv) expected model change where we label those points that would most change the current model, (v) expected error reduction where we label those points that would most reduce the model's generalization error, and (vi) variance reduction where we label those points that would minimize output variance, which is one of the components of error. Figure 4 illustrates the two possibilities for assembling training data to train GEM classifiers.

**Figure 5:**
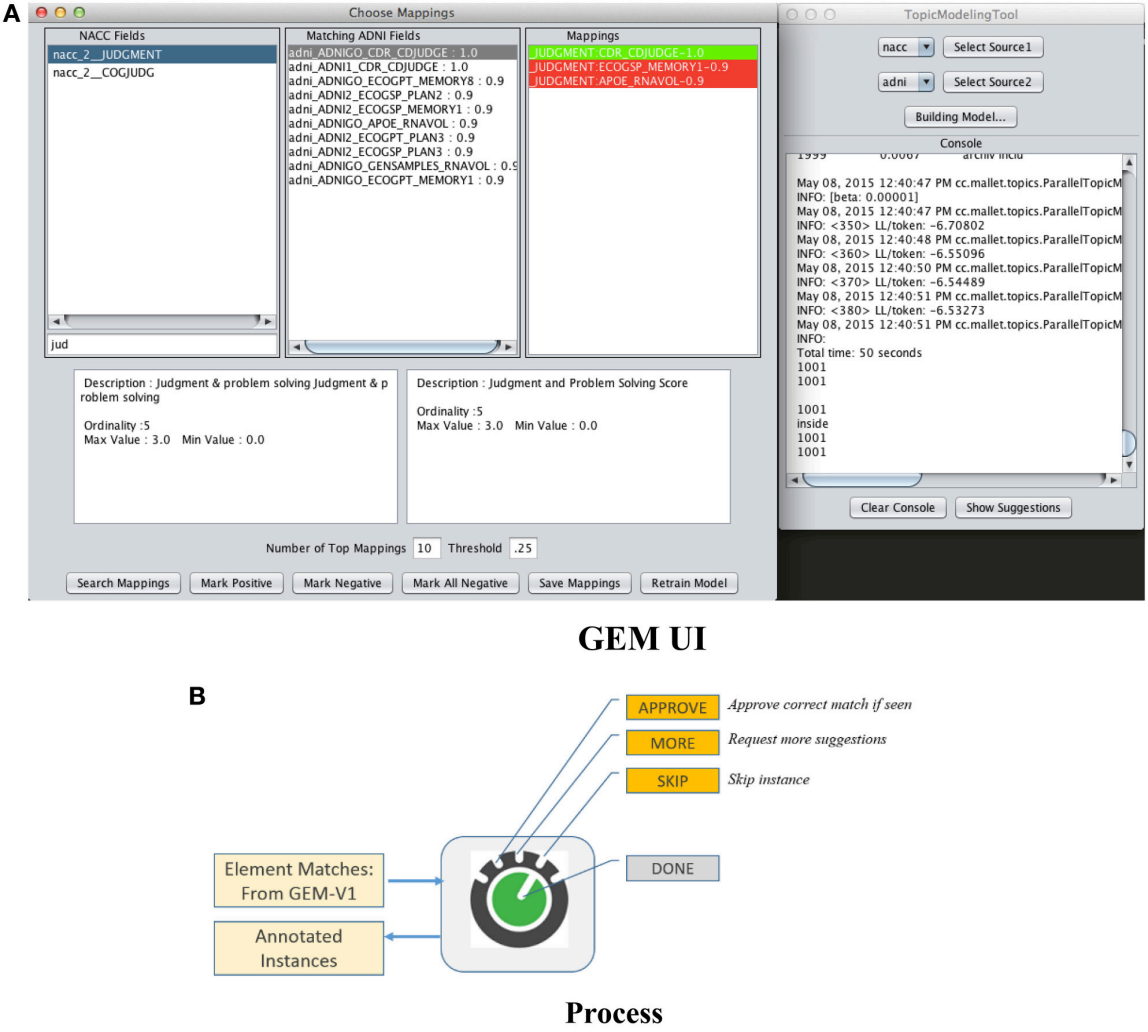
**GEM data mapping and active learning interface**. **(A)** GEM UI, **(B)** Process.

The first is “passive-learning” where a user must examine data dictionaries in a brute force manner and determine training instances. The second is to use active-learning where the system itself provides selected candidates for training instances, with the objective of significantly reducing user effort in determining good training instances. Figure 5A is a screenshot of the GEM data mapping user interface. Figure 5B illustrates the user options and workflow.

The user is provided a set of suggested matches for a data element as shown in Figure 5A. He or she can then (i) Approve a match if he can identify a correct match from within the suggestions, (ii) Ask for more match suggestions, (iii) Skip a particular training instance, and (iv) Finally choose to end the training data collection session. The UI also provides information about each suggested match such as its text description and associated metadata (not illustrated) to help the user determine if it is a match or not. The green highlight in the “Mappings” box in the UI in Figure 5A indicates that the user has vetted that element match as the correct one. The red highlights indicates match suggestions provided by the system that the user explicitly marks as incorrect.

### Estimating user effort

It is important to quantify the user effort with either passive-learning or active-learning, objectively. The effort is directly related to the number of data elements that a user must examine in determining a match and we thus employ the number of data elements examined as the matric. In active-learning, for a given source data element, the system suggests matches for that element in the order of the “rank” based on the text description match scores. Assuming that the user examines target elements in the order suggested by the GEM UI, the number of (target) elements examine per source element is simply the rank of the correct target match element. For instance if the rank of the correct target match is 9, the user would examine 9 elements from the UI in serial order and stop at the 9th element identifying it as a correct match. The total effort to determine *N* training instances is thus:

$$\begin{array}{l} \;E=\Sigma_{i=1\;to\;N}\;r\left(e_{i}\right)\\ where\;r(e)=rank\;of\;element\;e \end{array}$$

Estimating the effort for passive-learning, even in terms of the number of elements examined, is somewhat subjective. It depends on factors such as the user's familiarity with the domain, familiarity with the scientific concepts that the data elements represent, and also any prior knowledge of the particular dataset(s) to be mapped. We conducted an assessment where we tasked multiple developers—graduate students and data analysts to manually determine mappings across pairs of GAAIN datasets. The developers had varying degrees of familiarity with the Alzheimer's disease domain and datasets. We determined that the average number of target data elements examined per instance was approximately $\frac{N}{4}$ where *N* is the size of the target dataset (number of data elements). We will thus estimate the passive-learning effort in terms of this model, if the user ascertains *K* truth set mappings we estimate that the passive-learning mapping determination effort is $\frac{KN}{4}$.

### GEM system

As a software system GEM is written in Java. It employs the following tools and libraries—(i) the H2 main memory relational database^5^, (ii) the Mallet toolkit^6^ for topic modeling and (iii) the Weka toolkit^7^ for machine-learning classification. The system works in a scalable fashion on a regular desktop or laptop computer with Java being the only requirement. We are currently working on (a) making the GEM software available for use free and open-source to the community and (b) providing the GEM data mapping capabilities as a service that can be invoked over an API.

## Results

We conducted a series of experimental evaluations with GEM-2.0 system, which are centered on evaluating the mapping accuracy with machine-learning classification added, and also the effort in training the system for mapping.

### Experimental setup

The experimental evaluations have been conducted on data mapping across various GAAIN datasets from different institutions. We used six of the data sources of Alzheimer's disease data that we have in GAAIN namely (1) the Alzheimer's Disease Neuroimaging Initiative (ADNI) (Mueller et al., 2008), (2) the National Alzheimer's Coordinating Center database (NACC) (Beekly et al., 2007), (3) the Dominantly Inherited Alzheimer Network database (DIAN) (Morris et al., 2006), (4) the Integrated Neurogenerative Disease Database (INDD) (Xie et al., 2011), (5) the Layton Aging and Alzheimer's Disease Center database (LAADC) (Wu et al., 2013), and (6) the Canadian Longitudinal Study of Aging (CLSA) (Raina et al., 2009). The data providers provided us with data dictionaries for all of these sources. We manually created truth sets of data mappings across these dataset pairs, which are used as the gold standard against which GEM generated mappings and training effort are evaluated. Each of these datasets contains between five and then thousand distinct data elements. On an average, between any two dataset pairs from the above, we have about 200 elements that correspond across both datasets. The duration to determine these mappings manually was about 3 days per pair on average, and with the actual time effort ranging between 8 and 12 h per pair. The mappings cannot be typically done in “one sitting” as the process may require seeking additional documentation or understanding of the data elements. The manual mappings were done by graduate students and data analysts with some familiarity of the neuro-informatics domain.

### Mapping accuracy with classification

**Figure 6:**
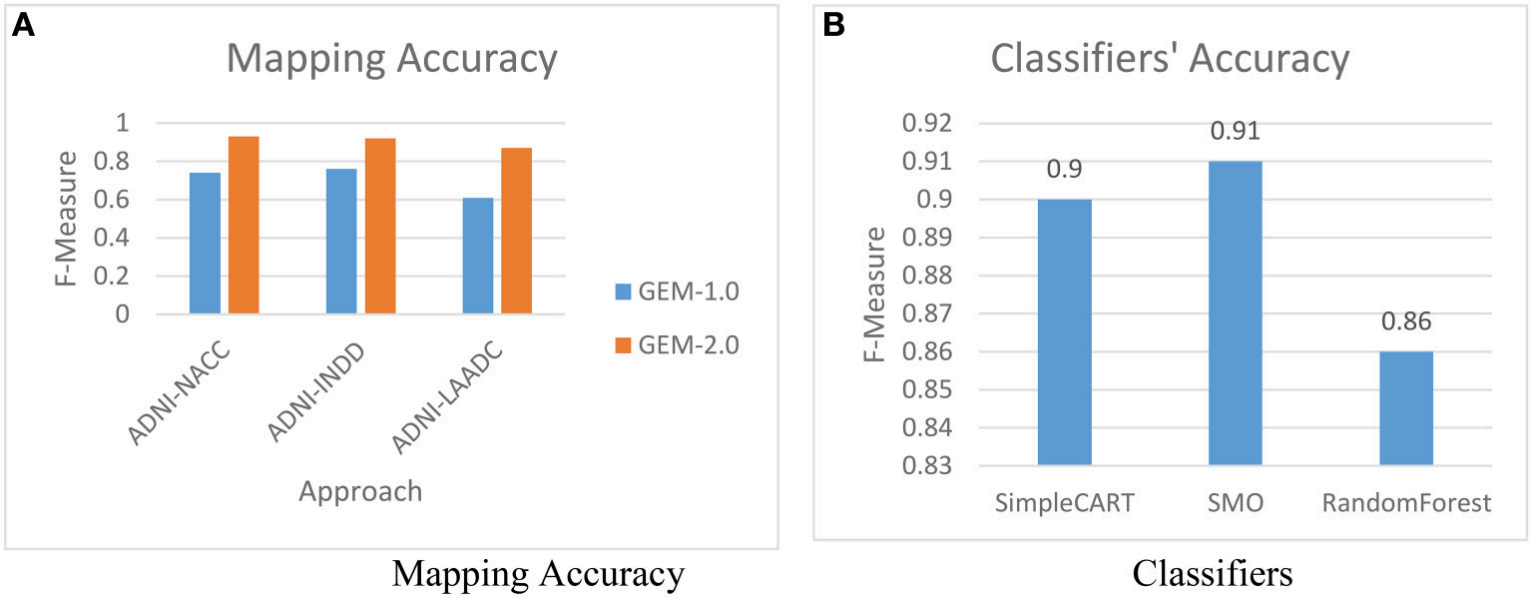
**Mapping accuracy**. **(A)** Mapping accuracy, **(B)** Classifiers.

The first experiment is to evaluate the impact of using GEM-2.0 (with machine-learning classification for matching) vs. GEM-1.0 (with no machine-learning classification). Figure 6A shows the mapping accuracies achieved, across various pairs of GAAIN datasets, with GEM-2.0 and GEM-1.0. The mapping accuracy is provided as F-Measure which is a metric that combines the precision as well recall—of the matches in this case. In pattern recognition and information retrieval with binary classification, precision is the fraction of retrieved instances that are relevant, while recall is the fraction of relevant instances that are retrieved (Baeza-Yates and Ribeiro-Neto, 1999). F-Measure itself is defined as: $F-Measure\;=\;2.\frac{precision.recall}{precsion+recall}$.

**Figure 7:**
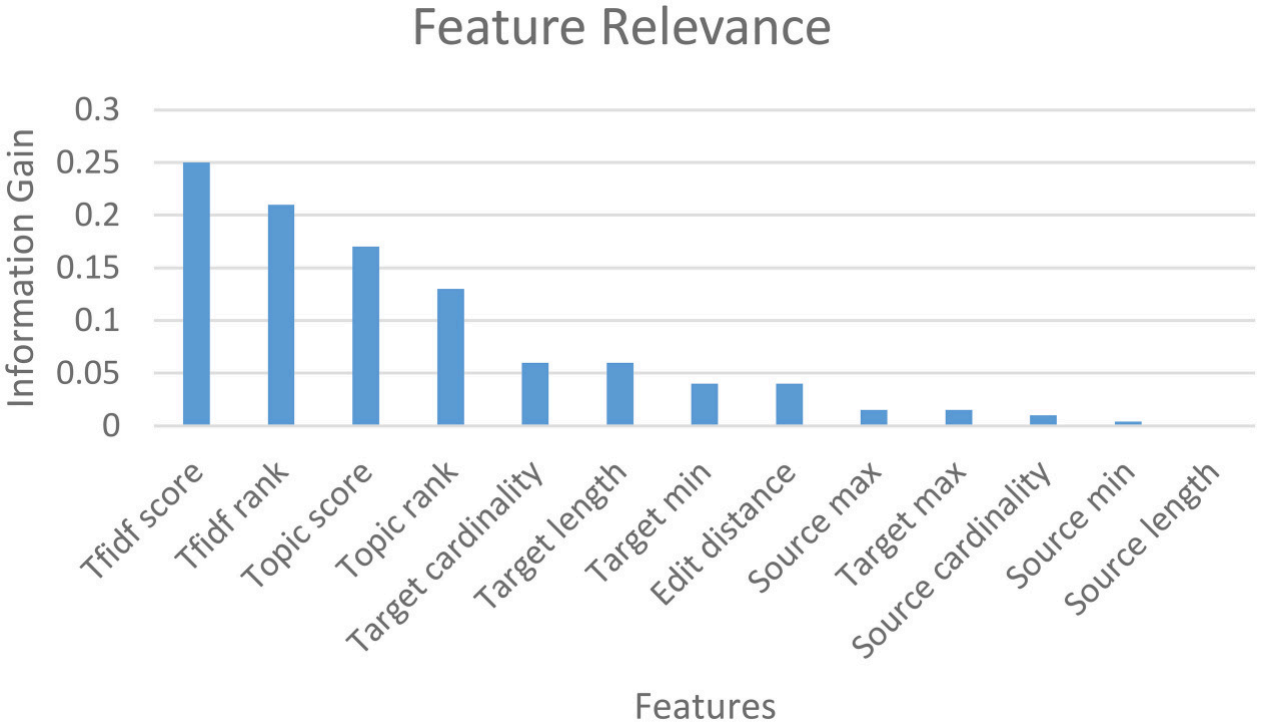
**Feature relevance**.

The mapping accuracy achieved with machine-learning classification in GEM-2.0 is high—at above 90% (with a suggested matches window size of 5) for all schema pairs (shown for only 3 pairs). GEM-2.0 is also significantly more accurate compared to GEM-1.0, thus establishing the importance of a classification approach over features. We evaluated multiple classifiers illustrated the best 3 in Figure 6B and determined that the hybrid SMO classifiers performs best for this task. We also analyzed the relative importance of the various features. Figure 7 lists the most important classification features in terms of the *information gain* associated with each feature. Information gain is an entropy based metric that essentially captures how discriminative (or not) an attribute or feature is for a classification task (Michalski et al., 2013). The information gain, while shown for one pair of schemas, is representative of the importance obtained for all other pairs. We see that the two text matching scores and ranks are the most important features driving the major part of the classification. We also observe that the TF-IDF based text match information is more informative compared to what the topic model match provides.

### Training effort with active learning

The objective of an active-learning capability is to make system training efficient. We assess the user effort in training the system with active-learning and provide a comparative estimate with the effort required with passive-learning. Table 2 illustrates the user effort, in terms of elements examined, for identifying truth set mapping sets, of increasing sizes. We show the effort for both active and passive learning (in terms of total number of elements that had to be examined). For active learning we employed two slightly different strategies for the user, namely: *Strategy 1*: For a given element, repeatedly request more suggestions until the correct match is shown and identified. *Strategy 2:* Skip the element if the matching element is not found in the initial set of suggestions. We also provide the mapping accuracy of the GEM system at each stage, i.e., after training it with the examples identified for the truth set.

**Table 2:** **Impact of active-learning on user effort**.

**Truth set size**	**Active learning effort (elements examined)**				Passive Learning Effort^*^ **(elements examined)**		**Accuracy (F-measure)**
	**Strategy 1**		**Strategy 2**				
	**Total**	**Average**	**Total**	**Average**	**Total**	**Average**	
10	342	34	213	21	2500	250	0.79
20	691	35	431	22	5000	250	0.84
30	1004	35	674	22	7500	250	0.91

* *Based on passive learning effort estimation*.

As Table 2 shows, the effort required is about 10 times more with passive-learning than with active-learning. The average number of target elements examined per source element is 250 elements with passive-learning whereas it is slightly above 20 elements on average for active-learning (Strategy 2). We also observe that a high mapping accuracy can be achieved with training the system with a modest number of training examples.

## Discussion

The objectives of the GEM system are similar to that of eMERGE (Pathak et al., 2011) which is a system for mapping phenotype data elements to standard data elements from ontologies such as the UMLS and SNOMED CT (Cornet and de Keizer, 2008). The eMERGE approach to mapping is purely knowledge driven, based upon simple lexical matches to ontology concepts in “pre-coordination” and “post-coordination” phases, depending upon whether the semantic terms have been previously seen and mapped or not, respectively. This approach is currently not applicable to the GEM environment where we may be mapping data across two dataset schemas vs. from a dataset to common data elements from ontologies. In the longer term however, as the GAAIN common model evolves and as we anchor GAAIN common model terms to standard data elements such as CDISC (Kuchinke et al., 2009), we could consider the addition of such semantic mapping techniques to GEM. The analysis on the element text descriptions in GEM is significantly more sophisticated. Further, GEM also exploits metadata details such as element codes, ranges of values etc., for mapping which eMERGE does not. Data mapping is, in general, frequently performed manually based on data dictionaries, on any other information such as database design diagrams (Doan et al., 2012, Principles of data integration) and in consultation with the original dataset creators and/or administrators. Data mapping is well understood (Doan et al., 2001, Reconciling schemas of disparate data sources: A machine-learning approach) and there are a number of software tools that have been developed in the past years that relate to it. Existing tools can be categorized as metadata visualization tools, Extract-Transform-Load (ETL), and schema-mapping tools. *Metadata visualization tools* are those that create a visual representation of the design of a database by examining the database itself. For instance SchemaSpy^8^ provides functionality of “reverse engineering” to create a graphical representation of metadata, such as an “ER” (Entity-Relationship) diagram (Garcia-Molina, 2008) from the database metadata. Altova^9^ is a tool for analyzing and managing relationships among data in data files in XML. These tools are relevant to our task as they can be employed to examine the data and/or metadata of new datasets. *ETL* tools provide support for data schema mapping. However, the mappings are not automated and have to be created by hand using a graphical user interface (GUI). Tools in this category include Talend^10^, Informatica^11^ and Clio (Halevy et al., 2005). The category most relevant to our data mapping problem is *Schema-Mapping* which provides automated mapping of data elements from two different database or ontology schemas. These tools take as input the data definition language or “DDL” (Garcia-Molina, 2008) associated with a dataset (database) and match elements across two database schemas based on the DDL information. Prominent examples in this category include the Harmony schema-mapping tool^12^ from the Open Information Integration or OpenII initiative and Coma++ (Aumueller et al., 2005). There are also schema-mapping tools that are based on “learning-from-examples,” i.e., the system is trained to recognize data element mappings from a tagged corpus of element matches (from the domain of interest). LSD (Doan et al., 2001, Reconciling schemas of disparate data sources: A machine-learning approach) is an example in this category. Another tool is KARMA (Gupta et al., 2012) which has more of an ontology alignment focus as opposed to data (element) mapping. KARMA has been employed for data mapping tasks in a variety of domains including in bioinformatics for mapping various drug databases to a common ontology, and also other domains such as geospatial data and environmental data. Finally, PhenoExplorer (PhenoExplorer, 2015), is an online tool that allows researchers to identify research studies of interest. Specifically, a researcher can search for studies along a set of dimensions, including race/ethnicity, sex, study design, type of genetic data, genotype platform, and diseases studied and the system determines the relevance of a study by mapping data elements in a study to dimensions specified by a researcher. Our work was motivated by the observation that the rich metadata available in data dictionaries of biomedical datasets can be leveraged toward a significantly more automated approach to schema-mapping than could be achieved with existing tools.

## Conclusion

We have described and evaluated the GEM-2.0 system in this paper. Compared to the existing state-of-the-art in schema mapping, the GEM system is better suited to and optimized for biomedical data mapping such as in Alzheimer's disease research. Our experimental evaluations demonstrate significant mapping accuracy improvements obtained with our approach, particularly by leveraging the detailed information synthesized for data dictionaries. The addition of machine-learning classification has helped us achieve significantly high accuracy of data mapping with real datasets. We have also demonstrated that with active-learning the system can be trained very efficiently with a very small number of training examples and minimal data element examination effort, to map data accurately in new datasets. Finally, the system and technology are applicable to data matching in any biomedical domain as the system is driven by biomedical data dictionaries not restricted to any domain or disease and can be trained with match examples specific to a domain of interest. GEM is currently also being applied to other data harmonization efforts in neuro-informatics but not restricted to Alzheimer's disease, for instance in a project on biomedical big data discovery where the focus is on developing a biomedical big data analysis infrastructure that is applicable to any biomedical domain or disease.

### Conflict of interest statement

The authors declare that the research was conducted in the absence of any commercial or financial relationships that could be construed as a potential conflict of interest.

## Footnotes

^2^ADNI—The Alzheimer's Disease Neuroimaging Initiative is a database of Alzheimer's disease research data created and maintained by the laboratory of Neuro Imaging.

^3^NACC—The National Alzheimer's Coordinating Center is another database of Alzheimer's disease research data.

^4^TFIDF, short for term frequency–inverse document frequency, is a numerical statistic reflects how important a word is for a document in a collection or corpus and is used as a weighting factor in information retrieval.

^5^http://www.h2database.com.

^6^http://mallet.cs.umass.edu.

^7^http://www.cs.waikato.ac.nz/ml/weka.

^8^http://schemaspy.sourceforge.net.

^9^http://www.altova.com.

^10^http://www.talend.com.

^11^http://www.informatica.com.

^12^http://openii.sourceforge.net.
